# Pregnancy and Neuromyelitis Optica Spectrum Disorder – Reciprocal Effects and Practical Recommendations: A Systematic Review

**DOI:** 10.3389/fneur.2020.544434

**Published:** 2020-10-16

**Authors:** Rohan D'Souza, Danielle Wuebbolt, Katarina Andrejevic, Rizwana Ashraf, Vanessa Nguyen, Nusrat Zaffar, Dalia Rotstein, Ahraaz Wyne

**Affiliations:** ^1^Division of Maternal and Fetal Medicine, Department of Obstetrics & Gynaecology, Mount Sinai Hospital, University of Toronto, Toronto, ON, Canada; ^2^Royal College of Surgeons in Ireland, University of Medicine and Health Sciences, Dublin, Ireland; ^3^Department of Obstetrics & Gynaecology, University of Ottawa, Ottawa, ON, Canada; ^4^Faculty of Medicine, University of Western Ontario, London, ON, Canada; ^5^Child Health Evaluative Sciences Program, Division of Paediatric Medicine, Hospital for Sick Children, University of Toronto, Toronto, ON, Canada; ^6^Division of Neurology, Department of Medicine, St. Michael's Hospital, University of Toronto, Toronto, Toronto, ON, Canada; ^7^General Internal and Obstetrical Medicine, Department of Medicine, Hamilton Health Sciences Center, McMaster University, Hamilton, ON, Canada

**Keywords:** neuromyelitis optica spectrum disorder, pregnancy, devic syndrome, systematic review, maternal and fetal risks

## Abstract

**Introduction:** Neuromyelitis optica spectrum disorder (NMOSD) is an inflammatory disorder of the central nervous system characterized by severe, antibody-mediated astrocyte loss with secondary demyelination and axonal damage, predominantly targeting optic nerves and the spinal cord. Recent publications have alluded to increased disease activity during pregnancy, and adverse maternal and fetal outcomes in patients with NMOSD. Our objective was to systematically review published literature to help counsel and manage women with NMOSD contemplating pregnancy.

**Methods:** We searched five databases including MEDLINE and EMBASE, for English-language publications describing pregnancies in women with NMOSD. Article selection, data extraction, and risk-of-bias assessment using Joanna Briggs' critical appraisal tool for case reports and case series, were performed in duplicate. Pooled incidences were calculated where possible, and a narrative summary was provided.

**Results:** Of 2,118 identified titles, 22 case reports and seven case series, representing 595 pregnancies in 389 women, were included. The mean maternal age was 28.12 ± 5.19 years. At least 20% of cases were first diagnosed during pregnancy. There were no maternal deaths. Pooled estimates for clinical outcomes could not be obtained due to inadequate reporting. NMOSD-related disability and relapses increased considerably during pregnancy and especially in the immediate postpartum period. Although a high proportion of early pregnancy losses were reported, an association with disease activity or therapeutic interventions could not be established. Apart from one publication which reported an increased risk of preeclampsia, there was no increase in adverse obstetric outcomes including preterm birth, fetal growth restriction or congenital malformations. Initial attacks and relapses were successfully managed with oral or intravenous corticosteroids and immunosuppressants, and refractory cases with immunoglobulin, plasma exchange and immunoadsorption.

**Conclusion:** Increased NMOSD-related disability and relapses during pregnancy the postpartum period may respond to aggressive management with corticosteroids and immunosuppressants such as azathioprine, which are safely administered during pregnancy and lactation. Emerging safety data on monoclonal antibodies during pregnancy, make these attractive options, while intravenous immunoglobulin, plasma exchange and immunoadsorption can be safely used to treat severe relapses. The complex interplay between NMOSD and pregnancy outcomes would be best understood through prospective analysis of data collected through an international registry.

**Disclosure:** Dalia Rotstein has served as a consultant or speaker for Alexion and Roche. She has received research support from Roche Canada. Rohan D'Souza has served as a consultant and speaker for Ferring Canada Inc and Ferring Global Inc, on topics unrelated to this manuscript. The other authors have no relevant relationships to disclose.

## Introduction

Neuromyelitis optica spectrum disorders (NMOSD) are inflammatory disorders of the central nervous system characterized by severe, immune-mediated demyelination, astrocyte loss, and axonal damage, predominantly targeting optic nerves and the spinal cord (1, 2). Unlike multiple sclerosis, which many believe to be primarily a cell-mediated disorder, NMOSD is thought to be primarily mediated by the humoral immune system, and is associated with a specific target antigen, the astrocytic water channel aquaporin-(3) (AQP4) (4). Circulating immunoglobulin-G antibodies (AQP4-IgG), which are now known to play a direct role in the development of NMOSD, have revolutionized the understanding of the condition (3), and have influenced the development of a new set of diagnostic criteria to define and further stratify NMOSD (1).

Women are more likely to be affected by seropositive (AQP4+) NMOSD than men, and in some series the ratio of women-to-men affected was as high as 9:1 (5). This gender disparity, the humoral basis of the condition, and the fact that NMOSD can affect those in the reproductive age group (median age of onset 32–41 years) (2), has generated much interest in NMOSD and pregnancy over the past decade, with a number of publications suggesting increased risk of relapse and greater disability during and immediately after pregnancy (6–13). Some others have also suggested an increased association between NMOSD and adverse pregnancy outcomes such as miscarriage and preeclampsia, especially in the presence of other autoimmune conditions (14). However, most publications, including multi-center studies, are limited by the small number of cases, making it difficult to interpret results and make firm conclusions.

The primary aim of this publication is to systematically review all published literature on pregnancy and NMOSD, with a view to determining the effect of the condition on pregnancy outcomes, and that of pregnancy on disease progression. The secondary aim is to explore management considerations, with a view to guiding clinical practice and future research.

## Materials and Methods

The study protocol was registered with PROSPERO (CRD42017055230) (15), and conducted and reported according to PRISMA (16) and MOOSE (17) guidelines, respectively.

### Data Sources and Searches

A medical information specialist conducted a literature search with the help of the study investigators, using the OvidSP search platform in MEDLINE, EMBASE, Web of Science, the Cochrane databases and PubMed in-process (for non-Medline articles, and those not yet indexed). A combination of subject headings and keywords was used to capture pregnancy (including pregnancy, pregnancy complications, obstetrics, and breastfeeding), various names for what now is known as NMOSD (including Devic syndrome/disease, neuromyelitis optica, NMO and NMOSD) and various terms used for anti-NMO antibody (including aquaporin-4 and AQP4), with articles included if indexed as of 23 October 2017. A more focussed search was repeated in March 2020 to include new publications. The search was limited to human data and restricted to the English language. No other restrictions were applied. The search strategy is presented as Supplementary Data 1. Additional articles were identified by scanning reference lists of included articles as well as excluded commentaries, editorials and review articles.

### Study Selection

#### Type of Studies

All prospective and retrospective studies reporting cases of NMO or NMOSD previously diagnosed, or diagnosed for the first time in pregnancy, were included. Given the rarity of the condition, we opted to include case reports and small case series, so as not to miss vital information with regard to disease progression and treatment modalities.

#### Types of Participants

We included all publications involving pregnant women with NMOSD, ideally diagnosed using the Updated Diagnostic Criteria (1). Given that these criteria were only revised in 2015, the diagnosis of NMO or NMOSD based on previous criteria (1, 18) were also included. Further, we have included cases based on the clinical phenotype. Therefore, patients were heterogenous with regard to AQP4 serotype, i.e. we included both seropositive and seronegative cases. Cases of multiple sclerosis and neurologic disorders mimicking NMO or NMOSD, or with uncertain diagnosis, were excluded.

### Outcomes

#### Maternal Outcomes

Maternal outcomes were maternal death, area postrema syndrome, details of neurologic presentation and progression including motor and sensory symptoms, spasticity, visual and hearing impairment, bladder or bowel dysfunction and seizures). We also made note of respiratory and cardiovascular symptoms, as well as obstetric outcomes including hyperemesis gravidarum, hypertensive disorders of pregnancy, gestational diabetes mellitus, antepartum and postpartum hospitalization including the need for admission to intensive care unit, mode of delivery, and labor and delivery complications such as postpartum hemorrhage or major perineal lacerations.

#### Fetal and Neonatal Outcomes

Fetal and neonatal outcomes included a miscarriage (fetal loss < 20 weeks), stillbirth (fetal loss >20 weeks), neonatal death (death within the first 28 days of life), growth restriction (weight <10th centile for gestational age), premature birth (birth before 37 weeks of gestation), admission to the neonatal intensive care unit (NICU), length of NICU stay, Apgar scores at birth and long-term neonatal outcomes if reported.

#### Treatment Outcomes

Treatment outcomes included details on treatment strategies and the maternal response to these strategies, including the involvement of multidisciplinary teams, peripartum obstetric and anesthetic management, management of obstetrical complications and emergencies, neonatal management, postpartum management of maternal symptoms and modifications to maintenance therapies.

### Data Extraction

A data extraction form was designed to include all available information on disease progression and the above pregnancy outcomes and pre-piloted. Two reviewers independently screened titles, abstracts and full texts, and disagreements were resolved through discussion, or through adjudication by a senior investigator, when disagreements persisted. Data from all included papers was extracted in duplicate and where clarification on interpretation of data was required, senior investigators with expertise in high-risk obstetrics and neurology, adjudicated. Data was extracted on year of publication, country and study setting; study design; number of pregnant persons and pregnancies; patient demographics and baseline characteristics; age at diagnosis of NMOSD; whether the patient had received another diagnosis prior to receiving the diagnosis of NMOSD; medical co-morbidities predating pregnancy and clinical status at onset of pregnancy; details of primary and secondary outcomes as outlined above; methods of identifying and controlling for confounders, if reported; methods of handling missing data if reported; and details on analysis, as presented. Although originally intended, based on the retrospective nature of most studies, and since information provided was sufficient to make decisions with regard to inclusion, we did not contact authors for additional information, as this was not likely to yield any more information than presented in the original manuscript.

### Quality Assessment

Since all included studies were either case reports and case series, to enable comparative scoring between studies, quality assessment was performed using Joanna Briggs' critical appraisal tool for case reports and series.

### Data Synthesis

#### Primary Analysis

Pooled incidences and 95% confidence intervals (CI) were planned for all maternal, fetal and neonatal outcomes, should the data have permitted this form of analysis. As considerable clinical and methodological heterogeneity between studies was anticipated, analysis was planned using DerSimonian-Laird binary random-effects meta-analyses on OpenMetaAnalyst® software (19). We planned on assessing statistical heterogeneity using *I*^2^ statistic, treating *I*^2^-values >75% as having a high degree of heterogeneity (20). Given the rarity of this condition, included studies were mostly case reports and case series with small numbers of patients and considerable heterogeneity between studies. For this reason, we primarily used tabulation and narrative synthesis in summarizing the data.

#### Subgroup and Sensitivity Analyses

Given the small-anticipated numbers of case series, we did not propose any a priori subgroup or sensitivity analysis. We aimed to assess publication bias using visual inspection of funnel plots with 95 and 99.7% control limits, in analyses where more than 10 studies were included.

## Results

### Included Publications and Pregnancies

**Figure 1:**
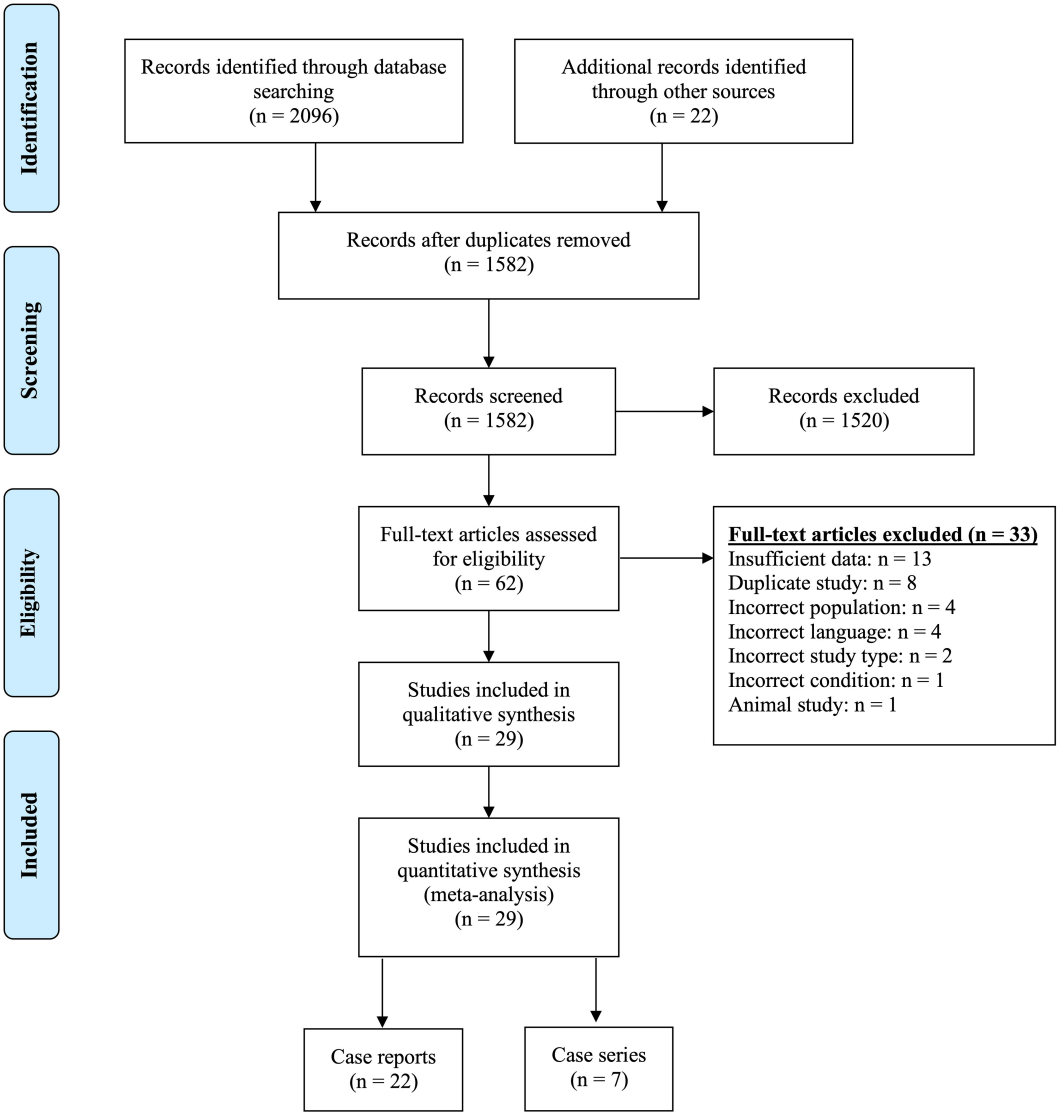
PRISMA diagram.

Our search identified a total of 2,118 titles and abstracts, of which 1,582 remained after removing duplicates. Following the first round of screening, 1,520 were found not to be relevant to pregnancy and NMOSD. We sourced the remaining 62 full-texts and excluded a further 33 were excluded for reasons identified in Figure 1 and described in Supplementary Table 2. Of the 29 included papers, 22 reported on individual cases [one pregnancy, a number of pregnancies in a single patient, or an account of all pregnancies in a number of patients] (21–42). The remaining seven publications summarized data on all pregnancies managed at one or more centres (6–11, 14).

### Characteristics of Included Pregnancies

The 22 case reports described 71 pregnancies in 54 women, and the seven case-series described 524 pregnancies in 335 women. Thus, this systematic review included a total of 595 pregnancies in 389 women with a diagnosis of NMOSD. The publications were mostly from Europe, the Americas and Asia, and the characteristics of included pregnancies are presented in Table 1.

**Table 1:** Characteristics of included publications and pregnancies.

	**Case reports**	**Case series**
Number of publications	22	7
Patients (pregnancies)	54 (71)	335 (524)
Geographical region		
• Europe	• 7/22	• 1/7
• North America	• 6/22	• 0/7
• South America	• 2/22	• 1/7
• Asia	• 6/22	• 3/7
• Multiple centers	• 1/22	• 2/7
Maternal age in years (mean ± SD)	28.12 ± 3.91	29.9 ± 5.19
Maternal ethnicity		
• Not reported	• 18/54 (33.3%)	• 153/197 (77.7%)
• Asian	• 23	• 38
• Black	• 7	• 4
• White	• 5	• 2
• Mixed	• 1	• 0
Gravidity	1.93 ± 1.41	1.63 ± 1.23^*^
Parity		
• Not reported	• 31	• 503
• Nulliparous	• 20	• 4
• Multiparous	• 21	• 17
NMOSD diagnosis	(denominator 71 pregnancies)	
• Diagnosed in index pregnancy	• 31	• 107/524
• Correct diagnosis prior to pregnancy	• 28	• Unclear
• Incorrect diagnosis prior to pregnancy	• 12	• Unclear
Diagnostic criteria for NMOSD met	43/71	524/524
• Aquaporin antibodies	• 65/71	
• Acute myelitis	• 38/71	
• Optic neuritis	• 23/71	
• MRI findings	• 31/71	
Medical comorbidities	(denominator 71 pregnancies)	Reported in 3/7 series and ranged from 12 to 63%
• Type 2 diabetes mellitus	• 1	
• Hashimotos thyroiditis	• 1	
• Sjogren syndrome	• 1	
• Systemic lupus erythematosus	• 2	
• Myasthenia gravis	• 1	
• Other autoimmune disease	• 1	

* *Only reported in two case-series; MRI, magnetic resonance imaging; NMOSD, Neuromyelitis optica spectrum disorder; SD, standard deviation*.

#### Demographic Details

The mean maternal age (during pregnancy) for all included patients was 28.12 ± 5.19 years. Reporting of patient demographics was limited, especially in the case series. For example, maternal ethnicity was not reported in one third of the case reports and for over three quarters of patients included in the case series, and we opted not to make assumptions with regard to ethnicity based on country of publication. Similarly, information on gravidity and parity was missing in most case series. Medical comorbidities were poorly reported in both case reports and case series. Where reported, the most common conditions included autoimmune disorders such as systemic lupus erythmatosus and Sjogren's syndrome, thyroid dysfunction, myasthenia gravis and antiphospholipid antibody syndrome. The reported demographic data are summarized in Table 1.

#### Diagnosis of NMOSD

In the case reports 31/71 (42%) described the diagnosis of NMOSD being made during the index pregnancy, while 28/71 were diagnosed as NMOSD prior to pregnancy, and in 12/71 (17%) cases, an alternate diagnosis (multiple sclerosis, transverse myelitis or neurosarcoidosis) made prior to pregnancy, was changed to NMOSD during pregnancy, but did not affect treatment decisions during pregnancy. Case series described 107/524 (20%) *de novo* diagnosis of NMOSD in pregnancy, but were unclear in their reporting of diagnoses made prior to pregnancy. Where reported, the average age at diagnosis of NMOSD for the entire cohort, was 31.49 ± 7.41 years (for case reports alone, 29.9 ± 5.91 years). While the case series confirmed that criteria for NMOSD diagnosis were met in 100% of cases, details on the specific criteria based on which the diagnosis was made, were lacking. Case reports on the other hand, provided greater detail on the specific criteria being met, in terms of AQP4 antibodies (65/71), clinical symptoms (61/71) and MRI findings (31/71).

### Outcomes

#### Maternal Outcomes

##### Maternal Medical Outcomes

The most commonly reported maternal neurologic signs and symptoms reported during pregnancy included sensory abnormalities including dysesthesias, paraesthesias, hypoesthesia, allodynia, and neuropathic pain (29 episodes in 16 pregnancies, between 9 weeks' gestation and 2-weeks postpartum), motor weakness (22 episodes in 10 pregnancies, occurring between 9 weeks and 2-months postpartum), visual symptoms (17 episodes in 10 pregnancies, occurring between 9 and 34-weeks of gestation), bladder and/or bowel incontinence (10 episodes in 6 pregnancies, occurring between 9 and 34 weeks' gestation) and spasticity (five episodes in five pregnancies, between six and 34 weeks of gestation). In addition, there were three reports of “features of transverse myelitis” without specifying signs or symptoms, between the first trimester and 10-days postpartum, two reports of severe respiratory symptoms (dyspnea requiring oxygen therapy as part of a relapse that also involved severe spastic tetraparesis and widespread sensory disturbances, and acute respiratory failure requiring intubation and mechanical ventilation), and one of seizures, although no further details on the seizures were provided. There were no maternal deaths or gait abnormalities.

##### Disability

The dramatic progression of NMOSD-related symptoms often results in considerable disability during pregnancy, which has been quantified as Expanded Disability Status Scale (EDSS) scores, that range from 0 (normal) to 10 (death by the disease) and increase in degrees of 0.5 points. Bourre et al. noted a considerable increase in the EDSS score from 1.5 ± 1.7 to 2.6 ± 1.9, *p* = 0.027), suggesting that pregnancy might have a greater effect on disability in NMOSD than in multiple sclerosis (10). Huang et al. reported a statistically significant increase in EDSS scores from 1.55 ± 0.38 before conception to 1.93 ± 1.41 during pregnancy, and 2.88 ± 2.14, in the postpartum period. Fragoso reported an increase in EDSS scores from 1.33 ± 1.60 before pregnancy to 3.01 ± 1.83 a year after childbirth (*p* = 0.06) (7). In summary, 42% of cases had increased EDSS scores during or soon after pregnancy (6).

##### Maternal Obstetric Outcomes

The only antenatal obstetric outcome reported was that of hypertensive disorders of pregnancy including preeclampsia, which affected 17/146 (11.6%) pregnancies (7, 14, 21, 23, 39). It must be noted that only two case series and eight case reports commented on this outcome. Two of these developed eclamptic seizures during pregnancy. There were limited data on the mode of initiation of labor (spontaneous vs. induced) or the use of labor analgesia. In the 100 instances, where the mode of delivery was reported, most (60%) had vaginal births. Where cesarean deliveries were undertaken, limited data were presented on their indication. The gestational age at delivery was only mentioned in 37 pregnancies, of which 7 (19%) occurred preterm (before 37 weeks of gestation). Two of these were vaginal births at 35 weeks' gestation, with no mention on whether they occurred spontaneously or were medically induced. Of the other five, one was induced at 31 + 3 weeks following the diagnosis of intrauterine fetal death; two preterm cesarean deliveries were performed for obstetric indications (severe preeclampsia at 25 weeks and fetal well-being concerns at 33 weeks); and two cesareans were performed at 32 and 35 weeks in view of refractory neurological symptoms (respiratory symptoms in one, and progressive weakness and blindness in the other), despite treatment. A mention was made of one patient presenting in very advanced labor, on account of not feeling uterine activity.

##### Relapses

Annualized relapse rate (AAR), which refers to the number of relapses per patient and per year has often been used to describe relapses in patients with NMOSD, including during pregnancy and in the postpartum periods. Studies have suggested increased risk of relapse and greater disability during and immediately after pregnancy (6–8, 10), especially in those not on immunosuppressive treatment at the time of conception (9). With regard to the antepartum period, it is unclear whether relapses occur with greater frequency during any particular trimester. Tong et al. reported no increase in relapses during pregnancy in 234 pregnancies (11). Fragoso et al. reported that relapses were most common in the first trimester (7), while Bourre reported it to be highest in the third trimester (10). Huang et al. reported a 0.44-times decrease in relapse in the third trimester when compared with the year before conception (6). It is possible that these variations depend not just on the natural course of the disease, but also upon the use of suppressive medications, and/or the ARR prior to conception. With regard to the postpartum period, most studies reported an increased relapse rate in the first few months following childbirth, but there is no consensus on whether the relapse rate stabilized within 6 months (6, 10–13). Of the postpartum relapses described in the literature, most occurred within the first 3 months postpartum. Relapses were described as early as within 7–10 days, and as late as 17–30 months following childbirth, which are unrelated to the course of pregnancy. Eight studies reported no relapse during the study follow up period, which when described, ranged between 3 months and 2 years. In addition to the stage of pregnancy, there seems to be a positive correlation between relapse rates and seronegative AQP4-IgG status [OR 3.84, *p* = 0.025)], the presence of other autoimmune conditions or antibodies [OR 2.48, *p* = 0.025)] and those receiving no treatment during remission [OR 1.19, *p* = 0.025)] (6). The lack of immunosuppressive treatment was identified as a risk factor for relapses in several studies (9, 11, 39) while factors that were not found to be correlated with relapses included age at onset of NMOSD (6), maternal age at pregnancy (7), presence of initial symptoms (6), pre-pregnancy relapses (7), regional analgesia/anaesthesia (7, 10) or breast feeding (10). Information on the effect of race or mode of delivery on relapse rates was insufficient to draw conclusions.

#### Fetal and Neonatal Outcomes

##### Mortality Outcomes

Data on pregnancy loss were explicitly presented for 531 pregnancies, of which 139 pregnancy losses occurred prior to viability (spontaneous miscarriages or pregnancy terminations on account of the condition or medications), and two were stillbirths. The trimester/ gestational age at pregnancy loss was only presented in 12 instances, eight of which were in the first trimester, three in the second and one in the third trimester. The temporal association between exacerbation in the maternal medical condition and fetal loss, was mentioned in three instances—two miscarriages following episodes of transverse myelitis requiring treatment with high-dose steroids and plasma exchange, and one stillbirth at 31 + 3 weeks concurrent with seizure activity in the mother. For the remainder of the pregnancy losses, temporality could not be ascertained. Data were also lacking in most instances, on the proportions of pregnancies that were lost spontaneously vs. those that were terminated, and the reasons for terminations.

##### Fetal Growth Restriction and Preterm Birth

There were three reported cases of fetal growth restriction in two publications (38, 39). However, birth-weight centiles based on gestational age could only be calculated for eight publications that provided details on birth weight, and fetal growth restriction could be confirmed only in one case (1,635 g at 33 weeks' gestation, which is under the 3rd centile) (38). Of the 98 pregnancies for which data on gestational age at birth was available, there were 12 reported preterm births (under 37 weeks' gestation). Of these, one followed preterm premature rupture of membranes at 36 weeks, three others occurred at 35 weeks, and the gestational age for four presumably spontaneous births was not known. The other four occurred between 25 and 33 weeks of gestation. In two of these cases, labor was induced (severe preeclampsia at 25 weeks and intrauterine fetal death at 31 + 3 weeks) and two cesarean deliveries were performed at 32- and 33-weeks' gestation, for uncontrolled maternal symptoms and suspected fetal growth restriction, respectively (7, 23, 27, 39).

##### Neonatal Outcomes

There were no reports of neonatal deaths. Six case reports presented Apgar scores at birth, to indicate the condition in which the baby was born. Besides the preterm infants that were admitted to the NICU, neonatal admissions were also described for five other infants, for transient myasthenia gravis in the absence of AQP4-Ig antibodies, which responded to intravenous immunoglobulin (IVIg) treatment but required prolonged hospitalization (25 days) (31), third-degree congenital heart block treated with intravenous dexamethasone (in a mother who had anti SS-A and anti SS-B antibodies) (33), hydrocephalus (14), congenital anomaly (aplastic left lung and fusion of digits) and seizures (40), and an unknown indication (7 days) (7). Congenital malformations, or their absence, were explicitly reported in 10/28 publications, while an additional 10 reported on a healthy newborn, presumably without any anomaly and with an intact neurological examination. A normal neurological examination was explicitly mentioned in five publications, four of which also described the AQP4-IgG titres/ levels at birth. Two of these publications, also described levels at follow-up, which in one case dropped to one-quarter of the original levels in 8-weeks (26), and the other wherein titres of 1:100 normalized over 6 months (28). Two studies described infant follow up ranging from 14 months to 18 years (7) and 6 months to 12 years (9), respectively.

### Management Strategies

All publications provided details on management strategies during pregnancy, and to some extent, the response to these strategies.

#### Multidisciplinary Team

Eleven publications explicitly described the involvement of a multidisciplinary team, mostly involving a neurologist or internal medicine physician and an obstetrician, but in three instances each, also involved anaesthesiologists and neonatologists. Where multidisciplinary team involvement was not explicitly mentioned, three publications were authored by a team involving neurologists and obstetricians, with one each additionally co-authored by an anaesthesiologist and ophthalmologist. Seven publications were authored by neurologists alone, one by obstetricians and in nine instances, the team of physicians was unreported.

#### Medical Management of Symptoms

Medical management was not always described in detail, especially in case series, which tended to focus more on disability and relapse rates during pregnancy. Where described, 29/74 (39%) pregnancies did not receive any medical management. When treatment was administered, oral corticosteroids and immunosuppressive agents formed the mainstay, both for prophylaxis against relapses, as well as for the initial management of relapses. The immunosuppressive agents of choice were azathioprine (35 pregnancies), tacrolimus (7 pregnancies), cyclophosphamide (2 pregnancies) and methotrexate (2 pregnancies). Neuropathic pain was most commonly managed with agents such as gabapentin, amytriptiline and clonazepam, and painful spasticity with baclofen.

#### Management of Relapses

The initial management of relapses involved high-dose corticosteroids and/or the introduction of immunosuppressive agents, as described above. In addition, the use of intravenous corticosteroids was described in 15 pregnancies, 14 of which used methylprednisone, while one described the use of intravenous dexamethasone (5 mg/day for 5 days) to treat sphincter disturbance. The use of plasma exchange, with no adverse pregnancy events, was described in 12 cases, with as many as 24 sessions, until resolution of symptoms. The use of IVIg was described in six pregnancies, with one publication (6), suggesting lower birth weight of neonates of those treated with intravenous methylprednisone and/or IVIg during pregnancy (2,444 ± 440 vs. 3,060 ± 466 g, *p* = 0.002). However, this paper did not adjust for confounding variables such as maternal comorbidities, placental insufficiency, fetal growth restriction and prematurity. The use of biologics (rituximab) was described in 15 pregnancies, but treatment in all cases was deferred until after childbirth, or initiated in the postpartum period. In those on biologics prior to pregnancy, biologics were often withheld until childbirth, and re-introduced in the postpartum period. The use of postpartum mitroxantrone was described in one case, along with corticosteroids. It must be noted that in many instances, patients had concurrent autoimmune conditions which may have warranted the above treatments.

#### Labor Analgesia and Anesthesia

This was not described in most included publications. Where mentioned, regional analgesia and anesthesia (epidural and spinal) were successfully used. In general, while most anesthetists would avoid regional techniques in the setting of acute exacerbation of myelitis, there is no evidence to suggest a causal relationship between regional analgesia/anesthesia and onset of symptoms or relapses described by some (43), and therefore decisions should be individualized (44). This is particularly important since neuromuscular blockade administered as part of general anesthesia for cesarean deliveries is associated with a risk of aspiration and respiratory muscle weakness (44).

### Risk of Bias (ROB) Assessment

**Figure 2:**
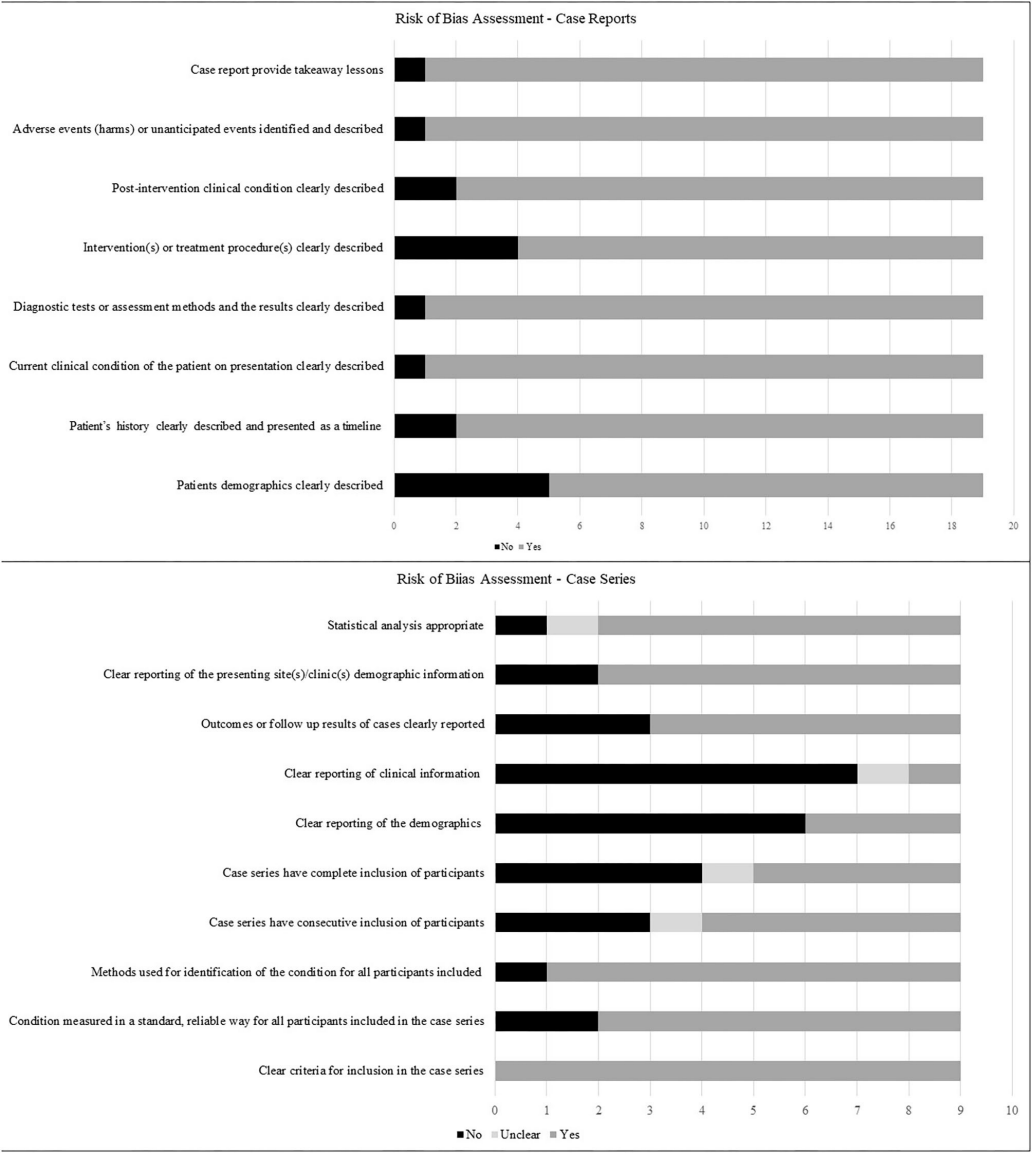
Risk of bias of included studies describing the proportions of studies fulfilling the criteria for case reports and case series as outlined in Joanna Briggs' critical appraisal tools.

ROB assessments for case reports and case series are presented in Figure 2. Case reports generally scored well on ROB assessment, with ~90% of them or greater, describing the patient's history, assessment methods, the clinical condition pre- and post-intervention, adverse events and take-away lessons. Patient demographics and interventions were described by 74 and 79% of the studies, respectively. ROB assessments for case series were less robust, with only the criteria for inclusion and the methods used for identification of the condition, clearly described in 100 and 89% of the series, respectively. In addition, 78% of series clearly reported patient demographics, performed adequate statistical analysis, and reported whether the condition was measured in a standard manner. In contrast, outcomes and follow up was adequately described in 67% of series, confirmation that cases were consecutive in 56%, complete inclusion of participants in 44% and patient demographics described in 33%, while clinical information regarding pregnancies was adequately reported only in 11% of the series.

## Discussion

This systematic review that included 22 case reports and seven case series described 595 pregnancies in 389 women with NMOSD. Despite inadequacies in reporting of pregnancy outcomes, the inability to determine the association between relapses and outcomes, or the effect suppressive treatment on preventing relapses and improving pregnancy outcomes, this review was able to confirm the following with regard to NMOSD and pregnancy.

First, pregnancy and the postpartum period are associated with increased NMOSD disease activity. There are a number of explanations for why pregnancy might accelerate the course of the condition, or the nature/frequency of symptoms. During pregnancy, the fetoplacental unit synthesizes Th2 cytokines, which induce downregulation of maternal Th1 cytokines that mediate cellular immunity, thereby increasing humoral immunity. This would imply that the disease activity of NMOSD (a Th2- mediated disease) should be considerably higher than that of multiple sclerosis, which many believe is primarily a Th1-mediated disease. However, a recent study has shown this not to be the case, suggesting that Th1/Th2 cytokine imbalance is not the primary pathophysiological pathway of NMOSD activity during pregnancy (11). It has also been suggested that the higher estrogen levels in pregnancy can lead to development of self-reactive peripheral B cells, which can increase antibody production in NMOSD (vs. multiple sclerosis which is not an antibody-mediated disease) (45). Although AQP4-IgG has been shown to cause placental inflammation and lead to negative pregnancy outcomes in animal studies, a recent study of the placentae of patients with NMOSD showed no clear decrease in placental AQP4 expression, no obvious placental inflammation or signs of damage in placental AQP4-IgG seropositive NMOSD patients, and no negative effects in term-born infants (46). It is possible that the increased disease activity and adverse pregnancy outcomes in patients with NMOSD is due to a multitude of factors, including the effect of pregnancy hormones such as estrogen, progesterone and glucocorticoids (11, 45). In fact, this review indicates that pregnancy and the postpartum period appears to be a high-risk time for disease activity and relapses. This is particularly true in the immediate postpartum period, where initiation or augmentation of immunosuppressive therapy might offer an opportunity for reducing relapses. In addition, disease activity might also be increased during the course of pregnancy, and increased disease activity may be associated with worse pregnancy outcomes. This suggests a role for immunosuppressive therapy to reduce disease activity and prevent relapses.

Second, although no maternal deaths have been reported, relapses are associated with considerable disability, both during and after pregnancy, which again may be amenable to the prompt initiation or increasing the dose of pre-pregnancy immunosuppressant medication. The commonest neurologic abnormalities occurring during pregnancy were sensory, although motor weakness, spasticity, visual symptoms, sphincter disturbances and serious respiratory morbidity were all reported.

Third, maternal obstetric outcomes may be no different from the general population. Although difficult to deduce the exact incidence of conditions from case reports and case series, especially when most did not report on obstetric conditions, it seems like the incidence of spontaneous preterm births are no greater in patients with NMOSD than with the general population. The one study which provided detailed information on preeclampsia, reported a higher rate [11.5% (6.27–18.9%)] than in population studies, and higher odds in women with other autoimmune disorders or prior miscarriages (14). However, NMOSD was not identified as an independent risk factor for preeclampsia. Based on this limited data, and given that the definition of preeclampsia has changed considerably over time, it would not be possible to conclude that the incidence of preeclampsia is truly increased in those with (or as a consequence of) NMOSD.

Area postrema syndrome, which refers to attacks of intractable nausea, vomiting, or hiccups, in the context of a lesion in the dorsal medulla, occurs in ~30% of patients with NMOSD and must be differentiated from hyperemesis gravidarum or severe nausea and vomiting in pregnancy, which occurs in ~1% of pregnant women (47). Although there is considerable overlap between the two, hyperemesis gravidarum often does occurs exclusively in the first half of pregnancy and may be associated with liver enzyme derangements and abnormalities in thyroid function testing, both of which would not be typical of area postrema syndrome. If in doubt, a brain MRI should be performed with any new acute presentation of severe vomiting in a woman with NMOSD. Identification of a lesion in the dorsal medulla would support the diagnosis of area postrema syndrome of NMOSD. Of course, it is more challenging if this is the first presenting sign of NMOSD in a pregnant woman. Area postrema syndrome usually responds well to high-dose corticosteroid therapy.

The vast majority of pregnancies resulted in vaginal birth, although some cesarean deliveries were undertaken on account of disease activity. Unless clinically indicated for fetal or maternal reasons, cesarean delivery is not required in those with NMOSD. No conclusions could be drawn with regard to the effect of the mode of delivery on the postpartum course. Although there are theoretical concerns that pre-existing demyelinated neurons may be more susceptible to neurotoxicity from local anesthetic agents, general anesthesia, in addition to its pregnancy-related risks also carries the risk of increased neuromuscular junction responses to muscle relaxants in those with NMOSD. Decisions on the choice of anesthesia should be individualized and involve shared decision-making with a multi-disciplinary team (48).

Fourth, the high fetal loss rate reported cannot be definitively attributed to NMOSD disease activity. Many series did not distinguish between pregnancy loss due to spontaneous miscarriage vs. pregnancy termination, and even when they did, it was difficult to determine whether spontaneous miscarriages, which are not uncommon even in healthy pregnancies, were the consequence of increased disease activity, co-existing autoimmune conditions or medications. Based on information provided, there was no increase in rates of congenital malformations, fetal growth restriction, stillbirths, or neonatal deaths. Neonates were delivered in good condition, although detailed neurological examinations were not provided. AQP4-Ig levels in cord blood were reported only in a small number of pregnancies. When reported, levels tended to return to normal within 6 months.

Fifth, a condition as rare as NMOSD is unlikely to be encountered by many healthcare professionals, and multidisciplinary input that includes neurologists, internal medicine physicians, high-risk obstetricians, ophthalmologists, anaesthesiologists and neonatologists is vital to optimize outcomes for mother and baby.

Sixth, pharmacologic management of NMOSD in pregnancy is highly variable and targets disease modification or symptom relief. It can range from supportive management with close observation to oral and intravenous corticosteroids (pulse and maintenance), various immunosuppressive treatments, IVIg, plasma exchange, and supportive treatment for symptoms. Although, after careful discussion of risks and benefits, and the knowledge that symptoms of NMOSD often worsen in pregnancy, an approach involving conservative (unmedicated) management may be an option for those with stable disease activity (22, 28), emphasis should be placed on the safety of many immunosuppressive treatments during pregnancy and while breastfeeding. This review shows that although relapses were managed aggressively, 39% of pregnancies were not on any medications during pregnancy. It is unclear whether this is the result of a general reluctance to administer medications during pregnancy, and whether the lack of suppressive treatment with steroids/ immunosuppressants could explain the high relapse rates. Initiation of prophylactic immunosuppressive treatment or increasing the dose of existing medication during pregnancy and in the early postpartum period could prevent relapses. A detailed account of therapeutic considerations with NMOSD and pregnancy has been recently published (49). A summary of various medications and their safety during pregnancy and lactation, based on most up-to-date evidence (49–53) is presented in Table 2, and discussed below

- Corticosteroids: Glucocorticoids are administered to patients with NMOSD both at high doses (1,000 mg/day for 5 days, administered intravenously) as a treatment for acute attacks and at lower doses (30 mg) as oral immunosuppressive therapy (49). Non-fluorinated glucocorticoids such as prednisone, prednisolone and methylprednisolone have a plasma half-life of 1–3 h and a duration of action of 12–36 h (49) Systemic corticosteroids are generally well-tolerated in pregnancy. Also, only 10% crosses into the fetal circulation due to placental metabolism and initial concerns with regard to their association with fetal orofacial clefts (58) has now been disproven (59–61). There may be a small association between the administration of corticosteroids and maternal obstetric outcomes such as gestational diabetes and hypertension, but in general the benefits in pregnancy outweigh risks. Lactation is compatible with glucocorticoid use, as glucocorticoid levels in breast milk are typically very low and no modifications to breastfeeding are recommended with short-term use. However, in those receiving high doses, delaying breastfeeding for 4 h theoretically would decrease the dose received by the infant (49, 52).
- Immunosuppressive Agents: Along with corticosteroids, other immunosuppressive agents form the mainstay of treatment of initial attacks and relapses. Azathioprine is a relatively safe option for use during pregnancy and lactation (49, 61), despite indications of a slightly increased risk of adverse outcomes, and should be initiated or continued, regardless of gestational age, should the clinical condition require pharmacologic management (62, 63). Tacrolimus has been used effectively, but is not among the first line treatments approved for NMOSD. Although associated with a low risk for congenital malformations (50) human studies suggest association with neonatal hypertension, hyperkalemia, and possibly prematurity (54–56). Cyclophosphamide is contraindicated for use in the first trimester and during lactation. Other drugs contraindicated during pregnancy and/or lactation included mycophenolate mofetil (MMF) and methotrexate due to a high risk of spontaneous miscarriage and congenital malformations, and mitoxantrone on account of ovarian toxicity resulting in permanent infertility, and substantial transfer in breast milk (61).
- Monoclonal antibodies: are being increasingly used in pregnancy. A recent systematic review of systemic autoimmune conditions showed that there is no association between their use during pregnancy and the risk of congenital anomalies or preterm deliveries compared with disease matched unexposed pregnant women (64). Owing to their high molecular weight, only small amounts are likely to be transferred into breast milk. These clinically insignificant amounts are also expected to be destroyed by proteolytic enzymes in the infant's gastrointestinal tract and, therefore, not absorbed into the bloodstream. Although women are generally advised not to breastfeed during treatment with monoclonal antibodies, this advice is likely to change in the near future. Rituximab crosses the placenta and induces a decrease in fetal B cell counts. However, this is reversible within 6 months of birth. Given during or after the second trimester, rituximab might lead to B cell depletion in the newborn baby, so B cell counts should be monitored in the baby and vaccinations planned accordingly. The concentration of rituximab in breast milk is found to be 240 times lower than in maternal serum (65). Eculizumab does not seem to have an adverse impact on pregnancy outcomes and umbilical cord blood concentrations are not sufficient to have a pharmacological effect on the fetus (66, 67). The drug has also not been detected in breast milk of mothers taking eculizumab, making it a potential treatment option in pregnant or lactating women with aggressive NMOSD disease. However, larger case series and long-term infant follow-up are required to further investigate the effects of eculizumab treatment during pregnancy and lactation. Studies on Tocilizumab suggest that there may be no increased risk of congenital malformations but a slightly increased risk of spontaneous miscarriage (25% vs. baseline risk of 12–15%) (68–72). Tocilizumab concentration in breast milk peaks on the third day after treatment administration, with a breast milk to maternal serum concentration ratio ranging from 1:500 to 1:1,000, and infants showing no signs of health problems, developmental delays or adverse events following routine vaccinations (73). Current phase-III clinical trials are ongoing on satralizumab (74) and inebilizumab (75), neither of which are expected to have teratogenic effects in humans, although pregnancy and lactation risks need to be further investigated.
- IVIg: is considered safe during pregnancy and lactation (76). The lower birthweight in those on IVIg reported in one publication (6), cannot be directly attributed to its use in pregnancy, and could be the result of other confounding variables, such as prematurity. Plasma exchange is not associated with increased risk of adverse effects during pregnancy and can be used after risk–benefit evaluation. General risks that include infection, coagulopathy, disturbances of electrolyte homeostasis, fluid shifts and hypovolemia need to be borne in mind. Immunoadsorption, wherein plasma is separated from blood cells, cleared of antibodies with an IgG-adsorbing column and reinfused, reduces the antibody burden more efficiently than plasma exchange. It is not known to be associated with clinically relevant adverse effects during pregnancy or lactation.

**Table 2:** Therapeutic recommendations for Neuromyelitis Optica Spectrum Disorder patients during pregnancy and breastfeeding.

**Medication**	**Pregnancy Risk** (50)		**Breastfeeding** **(****50**, **52**, **53****)**	
	**Teratogenicity (congenital malformation)**	**Other toxicity (Fetal/neonatal loss, prematurity, growth-and-developmental concerns)**	**Relative infant dose (RID)**	**Comment**
Corticosteroids	Human data suggests no increased risk of congenital malformations including orofacial clefts	Human data suggest no increased risk of fetal loss, but a possible association with preterm birth and low birth weight	Prednisone−0.35–0.53%; Prednisolone−0.09–0.18%	Compatible with lactation, especially with short term use. Suggest delaying breastfeeding for 4 h if on high doses
Azathioprine	Observational studies did not find a higher rate of birth defects in the offspring of women who received azathioprine therapy during pregnancy than in the general population	Exposure in the 3rd trimester has been linked to immunosuppression, and bone marrow suppression of the newborn has been reported, but modification of the dose in the 3rd trimester appears to reduce the risk of this toxicity	0.05–0.6%	Compatible with lactation. Suggest delaying breastfeeding for 4 h
Cyclophosphamide	Congenital defects when exposure occurs during organogenesis	Fetal bone marrow suppression is a potential toxicity when exposure occurs later in pregnancy	0.8% on day 1 to 0.9% on day 4	Reported cases of neutropenia and thrombocytopenia, and the potential for adverse effects relating to immunosuppression and carcinogenesis
Methotrexate	Methotrexate embryopathy	Exposure in second and third trimesters may be associated with fetal toxicity and mortality	0.5%	Contraindicated
Mitoxantrone	Animal studies do not suggest teratogenicity. However, due to its cytocidal effect on proliferating and non-proliferating human cells, its use is not recommended in the first trimester.	Toxic to some case reports suggest increase risk of spontaneous miscarriages and growth restriction	NA	Contraindicated
Mycophenolate mofetil	Human and animal data suggest risk. The use of mycophenolate mofetil (MMF) during early pregnancy is associated with major birth defects that may represent a characteristic phenotype	Associated with spontaneous miscarriages	NA	Limited information from few infants that have reportedly been breastfed with no adverse effects reported. Alternate drugs are recommended until more evidence is available.
Tacrolimus [Calcineurin inhibitor]	Human studies suggest low risk for congenital malformations, although animal studies indicate dose-related teratogenicity.	Animal studies indicated abortifacient properties in three species, but this has not been seen in human studies. Human studies suggest association with neonatal hypertension, hyperkalemia, and possibly prematurity (54–56)	0.06–0.5%	Compatible based on limited data
Eculizumab [Humanized monoclonal anti-C5 (terminal complement) antibody]	Case series suggest low risk of congenital malformations	Case series suggest no increased risk of fetal or neonatal loss	NA	Compatible based on limited data (57)
Inebilizumab	Evidence under review		NA	Evidence under review
Ocrelizumab	Evidence under review		NA	Limited data does not show harm-Evidence under review
Rituximab	Case series suggest no increased risk of congenital malformations	All human live births were healthy and none had structural anomalies that were thought to be related to rituximab	NA	Limited data does not show harm. Until more data available should be used with caution.
Tocilizumab [Humanized monoclonal anti-IL-6 antibody]	Case series and registry data suggest no increased rate of congenital abnormalities	Case series and registry data suggests no increased rate of spontaneous miscarriages	NA	Compatible based on limited data
Immune Globulin	No embryo-fetal risk attributable to immunoglobulin has been identified		NA	No human data-probably compatible
Plasmapheresis	Potentially safe in pregnancy		NA	Probably compatible
Gabapentin	Animal studies suggest congenital anomalies. Based on available human data, its use is recommended if benefits are deemed to outweigh risks.	Low birth weight, associated with increased risk of preterm birth and neonatal intensive care	2.34%	Limited human data-probably compatible
Amitriptyline	Occasional reports of congenital malformations but generally regarded as safe during pregnancy	Animal and human studies suggest no increased risk of fetal loss or other fetal toxicity	0.9%	Not expected to cause any adverse effects in breastfed infants, especially if the infant is older than 2 months. However, rare cases of sedation have been reported in neonates.

*DNA, deoxyribonucleic acid; NA, not available*.

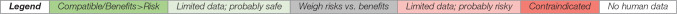

The safety of pharmacotherapy for NMOSD during pregnancy and lactation is summarized in Table 2.

This is the first systematic review on NMOSD and pregnancy, whose strengths include an exhaustive search strategy drawing on clinical data not only from case series but also case reports, to enable synthesis of as much information as possible. Despite the methodologic rigor of its conduct, it still has a number of limitations. First, the number of publications on NMOSD is limited, and data presented was insufficient to stratify relapses based on their nature, or draw firm conclusions with regard to ethnic variation, the effect of parity or comorbidities on disease activity, and whether disability and relapse rates are modified by pregnancy events, medications, trimester of pregnancy, use of regional analgesia, mode of delivery, or other pregnancy parameters. Second, although the inclusion of case reports added valuable information on disease progression, these publications are inherently biased, making it hard to determine incidences of various outcomes. Third, poorly and inconsistently reported outcomes as well as considerable heterogeneity between studies precluded any formal meta-analysis. Fourth, it is possible that some of the earlier case reports and series, all of which were retrospective, did not fully fulfill the revised diagnostic criteria for NMOSD. In particular, there were limited data on MRI findings, AQP4 antibodies and clinical symptoms, to determine whether the diagnostic criteria were met. Fifth, the lack of experimental studies in the area, made it difficult to make strong recommendations based on Grading of Recommendations Assessment, Development and Evaluation (GRADE) criteria. Finally, we recognize that the disease course and biology is driven by the serotype, AQP4 vs. myelin oligodendrocyte glycoprotein (MOG) vs. dual negative, rather than the clinical phenotype of NMOSD. However, serologic testing has changed considerably over time; MOG antibody testing was not widely available prior to around 2015, and was not widely reported in the included studies. Hence, some of the seronegative cases may have been MOG+ve, but there was no way of accurately guessing what number. The change in serologic testing as well as the poorer sensitivity of AQP4 testing in the past, makes it challenging to report findings based on the serotype, whether MOG or AQP4. Future research is needed to see if disease activity in pregnancy and postpartum differs by serologic status, and is beyond the scope of this review.

Despite these limitations, our systematic review adds to the growing body of literature on the pregnancy-specific risks to patients with NMOSD, key findings and recommendations of which have been presented in Table 3. Understanding the effect of pregnancy on NMOSD and vice versa, as well as the relationship between disease activity, relapses and treatment and adverse pregnancy outcomes, is critical to the management of NMOSD in pregnancy. Given the limitations of retrospective studies in determining temporality and guiding clinical practice, the initiation of an international prospective registry for pregnancy and NMOSD is strongly recommended, until which time, the findings of this systematic review may be used to counsel patients and encourage shared decision-making.

**Table 3:** Key findings and recommendations for NMOSD and pregnancy (modified from Mao-Draayer et al.) (49).

1. Pregnancy and the postpartum period, in particular, are associated with increased NMOSD disease activity and relapses. Initiation, continuation and/or augmentation of immunosuppressive therapy during pregnancy and in the immediate postpartum period should be considered to reduce attacks. 2. Although Aquaporin-4 (AQP4) is expressed at high levels in the placenta, and high pregnancy loss rates have been reported in NMOSD patients, especially in the first trimester, this review was not able to determine causality between NMOSD activity and spontaneous miscarriages, or comment on the influence of treatment on its risk. Similarly, apart from one publication which reported an increased risk of preeclampsia, there was no increase in adverse obstetric outcomes including preterm birth, fetal growth restriction or congenital malformations in patients with NMOSD. 3. Oral corticosteroids and azathioprine have proven safety for the treatment of initial attacks and relapses during pregnancy. In addition, high-dose intravenous corticosteroids, intravenous immunoglobulin, plasma exchange and immunoadsorption are safe and effective for the management of severe relapses in pregnancy. 4. There is emerging evidence on the safety of monoclonal antibodies such as rituximab. eculizumab and toclizumab during pregnancy and the postpartum period. Management should include monitoring of fetal growth by ultrasound, checking of neonatal B cell counts, and careful planning of newborn vaccination. 5. Mycophenolate mofetil, methotrexate and mitoxantrone are contraindicated, and should be discontinued prior to conception. Accidental administration during pregnancy warrants a discussion on teratogenic risks, and close follow up with ultrasound scans for structural anomalies and monitoring of fetal growth.

## Data Availability Statement

All datasets presented in this study are included in the article/Supplementary Material.

## Author Contributions

RD'S conceived the study, provided methodologic and content expertise, oversaw the analysis, and wrote all drafts of the manuscript. DW, KA, VN and NZ performed title and full-text screening and data extraction. DR provided input with regard to the interpretation of neurological symptoms and reviewed the manuscript. NZ and RD'S performed the analysis. RA reviewed the literature with regard to treatment options, helped with formatting and editing of the manuscript and helped with revising the manuscript. AW provided assisted with data extraction and writing up, and approved the final version of the manuscript. All authors contributed to the article and approved the submitted version.

## Conflict of Interest

The authors declare that the research was conducted in the absence of any commercial or financial relationships that could be construed as a potential conflict of interest. The reviewer ES declared a shared affiliation with one of the authors KA, and the handling editor is currently organizing a Research Topic with one of the authors DR. The review process met the standards of a fair and objective review.
