# Supplementary material for: Pregnancy and Neuromyelitis Optica Spectrum Disorder – Reciprocal Effects and Practical Recommendations: A Systematic Review
Source: Front Neurol. 2020 Oct 16;11:544434. doi: 10.3389/fneur.2020.544434 (PMC7596379; doi:10.3389/fneur.2020.544434)
Supplement: Supplementary file 1 [file Table_1.DOCX]

**Search Strategy for Neuromyelitis Optica**

**Medline Strategy**

Exp pregnancy/ or exp fetus/ or exp pregnancy complications/ or exp infant, newborn/ or exp postpartum period/ or exp prenatal diagnosis/ or preconception care/ or (pregnan* or obstetric* or gestation*).tw. or (f?etus or f?etal).tw. or newborn.tw. or (new adj1 born).tw. or neonat*.tw. or infan*.tw. or birth*.tw. or childbirth*.tw. or ( labo?r or puerper* or C?esar*).tw. or exp fetal development/ or exp fetal therapies or exp placental function tests/ or exp umbilical cord/ or exp prenatal diagnosis/ or exp fetal monitoring/ or exp perinatal care/ or exp obstetrical surgical procedures/ or exp anesthesia, obstetrical/ or exp analgesia, obstetrical/ or exp parity/ or exp apgar score/ or exp postpartum period/ or ((forcep* or vacuum or ventouse or instrument*) adj2 deliver*).tw. or (antepart* or ante-part* or prenat* or pre-nat* or perinat* or peri-nat* or peripart* or peri-part*).tw. or (postnat* or post-nat* or postpart* or post-part* or breastfe* or breast-fe* or (breast adj1 fe*)).tw. or obstetrics/

AND

Neuromyelitis Optica/ or Optic Neuritis/ or (devic or devic’s or devics).mp. or neuromyelitis optica.mp. or neuromyletis optica.mp. or optic neuritis.mp. or (NMO or AQ4 or AQP4 or AQ 4 or AQP 4 or AQ-4 or AQP-4 ).mp. or Aquaporin 4/ or (aquaporin adj2 “4”).mp.

**Embase Strategy**

Exp pregnancy/ or exp pregnancy disorder/ or exp pregnancy complication/ or exp pregnant women/ or exp newborn/ or (pregnan* or obstetric* or gestation*).tw. or (f?etus or f?etal).tw. or exp maternal care/ or exp puerperium/ or (newborn or (new adj1 born) or neonat* or infant*).tw. or (birth* or childbirth* or labo?r* or puerper* or c?esear* or episiotomy*).tw. or ((forcep* or vacuum or ventouse or instrument or vaginal) adj2 deliver*).tw. or (antepart* or ante-part* or prenat* or pre-nat* or antenat* or ante-nat* or perinat* or peri-nat or peripart* or peri-part*).tw. or (postnat* or post-nat* or postpart* or post-part* or lactat* or breastfe* or breast-fe*).tw. or (breast adj1 fe*).tw. or exp obstetric operation/ or exp anesthesia, obstetric/ or exp analgesia, obstetrical/ or exp parity/ or exp apgar score/ or exp postpartum period/or obstetrics/

AND

Myelooptic neuropathy/ or optic neuritis/ or (devic or devic’s or devics).tw. or neuromyelitis optica.tw. or neuromyletis optica.tw. or optic neuritis.tw. or (transverse adj1 myelitis).tw. or (anti-aquaporin or

anti-NMO or anti-neuromyelitis optica or anti-AQ4).tw. or aquaporin 4 antibody/

**Keyword Strategy for Web of Science, Cochrane and PubMed (in-process)**

(pregnan* or obstetric* or gestation*) or f?etus or f?etal or newborn or neonat* or infant* or birth* or childbirth* or labo?r* or puerper* or c?esear* or episiotomy* or forcep* or vacuum or ventouse or “instrument delivery” or “vaginal delivery” or antepart* or ante-part* or prenat* or pre-nat* or antenat* or ante-nat* or perinat* or peri-nat or peripart* or peri-part* or postnat* or post-nat* or postpart* or post-part* or lactat* or breastfe* or breast-fe*

AND

Devic or devic’s or devics or neuromyelitis optica or neuromyletis optica or optic neuritis or transverse myelitis or anti-aquaporin or anti-NMO or anti-neuromyelitis optica or anti-AQ4

Summary of Updated Search Results

Pregnancy and Devic Syndrome (Neuromyelitis Optica)

Inception to August 22, 2016, and re-run to October 23, 2017

Search Conducted October 24&25^th^

Numbers presented are after removal of duplicates

| **Database** | **Search Period** | **Results** |
| --- | --- | --- |
| Medline | 1946 – 23 Oct 2017 | 254 + 32 |
| Embase | 1947 to 23 Oct 2017 | 765 + 120 |
| Web of Science | To 23 Oct 2017 | 259 + 27 |
| Cochrane | To 23 Oct 2017 | 2 + 7 |
| PubMed in-process and non-medline | To 23 Oct 2017 | 50 + 66 |
| TOTAL |  | 1330 + 252 = 1582 |
