# Supplementary material for: Pregnancy and Neuromyelitis Optica Spectrum Disorder – Reciprocal Effects and Practical Recommendations: A Systematic Review
Source: Front Neurol. 2020 Oct 16;11:544434. doi: 10.3389/fneur.2020.544434 (PMC7596379; doi:10.3389/fneur.2020.544434)
Supplement: Supplementary file 2 [file Table_2.DOCX]

**Supplementary Table 2: Excluded studies**

| **Year** | **Author** | **Title** |
| --- | --- | --- |
| **Insufficient data for inclusion – conference abstracts unless otherwise stated (n=13)** | | |
| 2016 | Altintas A. | Outcomes of pregnancy in neuromyelitis optica patients: Turkish multicenter study data |
| 2014 | Mader S. | AQP4-igg placental transfer in NMO pregnancies |
| 2013 | Khoda T. | A case of patient with NMO who developed and relapsed at every pregnancy for three times |
| 2013 | Klawiter E. | High Risk of Initial Symptom Onset Post-Partum in Neuromyelitis Optica |
| 2013 | Liu Q. H. | Neuromyelitis Optica and Pregnancy |
| 2013 | Moinfar Z. | IL-6, AQP4 and CX43 modulations by estrogen in a model of astrocyte-microglia: Insight to the NMO and pregnancy outcome |
| 2013 | Shimizu Y. | Risk of neuromyelitis optica relapse associated with pregnancy on Japanese patients |
| 2012 | Yaguchi H. | Pathological study of subacute autoimmune encephalopathy with anti-AQP4 antibodies in a pregnant woman |
| 2012 | Lawin-O’Brien A. R. | Devic's neuromyelitis optica mimicking multiple sclerosis in pregnancy |
| 2011 | Garcia-Martin E.* | Devic neuromyelitis optica and bilateral internuclear ophthalmoplegia |
| 2010 | Protti A. | Optic neuritis in pregnancy heralding a neuromyelitis optica diagnosis |
| 2009 | Lana-Peixoto M. A. | Pregnancy may have a bad impact on devic's neuromyelitis optica |
| 2009 | Karim S.* | Devic's syndrome as initial presentation of systemic lupus erythematosus |
| **Duplicate data (n=8)** | | |
| 2014 | Nour M. M. | Pregnancy outcome in aquaporin-4 positive neuromyelitis optica spectrum disorder: a multi-center retrospective cohort study (Conference abstract) |
| 2014 | Nour M. M. | The impact of neuromyelitis optica spectrum disorder on pregnancy outcome (Conference abstract) |
| 2014 | Shimizu Y. | Risk of neuromyelitis optica spectrum disorder relapse associated with pregnancy on Japanese patients (Conference abstract) |
| 2014 | Shimizu Y. | Risk of neuromyelitis optica relapse associated with pregnancy on Japanese patients-part 2 (Conference abstract) |
| 2013 | Nishiyama A. | A case of neuromyelitis optica (NMO) which Rituximab was effective treatment in the acute phase (Conference abstract) |
| 2013 | Shimizu Y. | Relapse of Neuromyelitis Optica Associated with Pregnancy Is More Frequently Than That for Multiple Sclerosis (Conference abstract) |
| 2012 | Shimizu Y. | Neuromyelitis optica relapse associated with pregnancy: Similarities to multiple sclerosis (Conference abstract) |
| 2012 | Shimizu Y. | Neuromyelitis optica and pregnancy: Distinction from multiple sclerosis (Conference abstract) |
| **Non-English language (n=4)** | | |
| 2016 | Daouda M. T. | A treatment of neuromyelitis optica (Devic's disease) during pregnancy [French] |
| 2015 | Fernandez N. | Pregnancy and postpartum in a patient with neuromyelitis optica and systemic lupus erythematosus and literature review [Spanish] |
| 1965 | Vana V. | Contribution to the treatment of complete paralysis of respiratory muscles in neuromyelitis optica of Devic [German] |
| 1963 | Rousseau G. | Ecg changes during acute optic neuromyeutis [French] |
| **Incorrect population – non-pregnant population or diagnosis remote from pregnancy (n=4)** | | |
| 2016 | Hor J. Y. | A Natural History Study of the Effect of Pregnancy on Aquaporin 4 Antibody-Positive Neuromyelitis Optica Spectrum Disorder |
| 2015 | Chang T. | Gaze palsy, hypogeusia and a probable association with miscarriage of pregnancy--the expanding clinical spectrum of non-opticospinal neuromyelitis optica spectrum disorders: a case report. |
| 2010 | Srikugan L. | A case of optic neuritis and transverse myelitis in a postpartum Ghanaian female |
| s2010 | Zifman E. | Marked hypotonia in an infant of a mother with Devic disease. |
| **Incorrect study type – narrative reviews (n=2)** | | |
| 2016 | Zare-Shahabadi A. | Neuromyelitis optica and pregnancy |
| 2007 | Bencherifa F. | Devic's neuro-optic myelitis and pregnancy |
| **Incorrect condition (n=1)** | | |
| 2015 | Shimizu Y. | Management of multiple sclerosis and neuromyelitis optica in pregnancy and childbearing |
| **Animal study (n=1)** | | |
| 2013 | Saadoun S. | Neuromyelitis optica IgG causes placental inflammation and fetal death |
